# Mechanistic Studies of 1-Deoxy-D-Xylulose-5-Phosphate Synthase from *Deinococcus radiodurans*

**DOI:** 10.21767/2471-8084.100051

**Published:** 2018-01-29

**Authors:** Sumit Handa, Daniel R Dempsey, Divya Ramamoorthy, Nanci Cook, Wayne C Guida, Tyler J Spradling, Justin K White, H Lee Woodcock, David J Merkler

**Affiliations:** 1 Department of Chemistry and Biochemistry, University of California, San Diego, La Jolla, CA 92093, USA; 2 Departments of Medicine, Biological Chemistry and Molecular Pharmacology, Harvard Medical School and Brigham and Women’s Hospital, Boston, UK; 3 Department of Chemistry, University of South Florida, USA

**Keywords:** α-Carbanion/Enamine intermediate, Dimethylallyl pyrophosphate, Isopentenyl pyrophosphate, Non-mevalonate, Site-directed mutagenesis, TPP-dependent

## Abstract

The non-mevalonate dependent (NMVA) pathway for the biosynthesis of isopentenyl pyrophosphate and dimethylallyl pyrophosphate is the sole source of these terpenoids for the production of isoprenoids in the apicomplexan parasites, in many eubacteria, and in plants. The absence of this pathway in higher organisms has opened a new platform for the development of novel antibiotics and anti-malarials. The enzyme catalyzing the first step of the NMVA pathway is 1-deoxy-D-xylulose-5-phosphate synthase (DXPS). DXPS catalyzes the thiamine pyrophosphate- and Mg (II)-dependent conjugation of pyruvate and D-glyceraldehyde-3-phosphate to form 1-deoxy-D-xylulose-5-phosphate and CO_2_. The kinetic mechanism of DXPS from *Deinococcus radiodurans* most consistent with our data is random sequential as shown using a combination of kinetic analysis and product and dead-end inhibition studies. The role of active site amino acids, identified by sequence alignment to other DXPS proteins, was probed by constructing and analyzing the catalytic efficacy of a set of targeted site-directed mutants.

## Introduction

Isopentenyl pyrophosphate (IPP) and dimethylallyl pyrophosphate (DMAPP) are the precursors for isoprenoids, the largest family of biologically active compounds. The isoprenoid family of molecules represents the most diverse set of biochemical entities known and are of considerable biological importance. For example, cholesterol (an isoprenoid) serves as a precursor to the glucocorticoids, the androgens, the mineralocorticoids, the gestagens, and estrogen [1,2]. The biomedical importance of the isoprenoids has intrigued the scientific community on numerous levels for decades, with one of the earliest questions being: “How these molecules are synthesized in the cell?” This question was answered for cholesterol by Bloch, Cornforth, Lynen, and Popják in the 1930’s through the 1960’s [3–6]. The pathway defined by these researchers from acetyl-CoA to cholesterol is outlined in almost every undergraduate biochemistry textbook. A key intermediate in the canonical pathway of cholesterol and the isoprenoid biosynthesis is mevalonate.

For many years, it was thought that IPP, DMAPP, and all the biologically-occuring isoprenoids were derived solely from the mevalonate-dependent (MVA) pathway. An alternative pathway to IPP, DMAPP, and the isoprenoids was discovered by 1990’s, which accounts for isoprenoid production in the apicomplexan parasites, many eubacteria, green algae, and plants [7]. In this alternative pathway, IPP is not derived from mevalonate, but from pyruvate and glyceraldehyde-3-phosphate. A 5-carbon sugar phosphate, xylulose-5-phosphate is a key intermediate in the mevalonate-independent pathway of isoprenoid biosynthesis [7–9]. The enzymes catalyzing the reactions of the non-mevalonate dependent pathway (NMVA, also called the mevalonate-independent pathway or MEP) are completely different than the enzymes of the mevalonate dependent pathway (MVA). Many human pathogens produce their isoprenoids exclusively via NMVA or a combination of the MVA and NMVA, including *Plasmodium spp*, *Mycobacterium tuberculosis*, *Chlamydophila pneumoniae*, *Treponema pallidum.*

**Figure 1:**
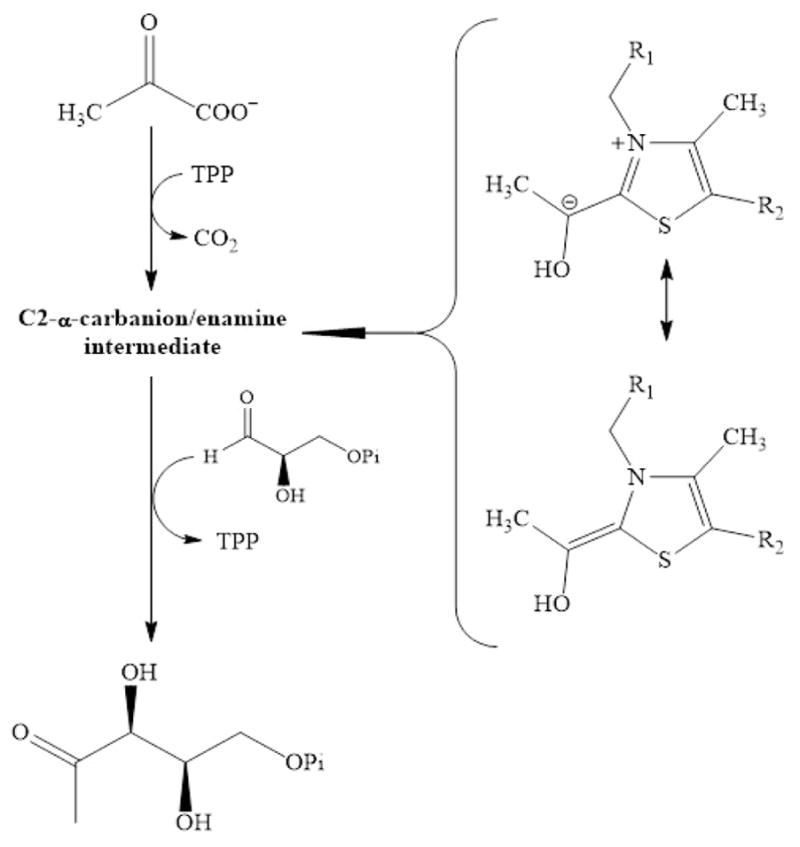
The reaction catalyzed by DXPS (R1=4′-amino-2-methyl-5-pyrimdyl and R2=β-hydroxyethyldiphosphate).

The first committed step in the NMVA involves the thiamine pyrophosphate- and divalent metal ion-dependent condensation of pyruvate (Pyr) and D-glyceraldehyde-3-phosphate (GAP) to form 1-deoxy-D-xylulose-5-phosphate (DXP) and CO_2_; as catalyzed by 1-deoxy-D-ylulose-5-phosphate synthase (DXPS) (Figure 1) [10]. DXP, the product of the DXPS reaction, is used not only for the production of IPP, DMAPP, and the isoprenoids, but also for the biosynthesis of thiamin (vitamin B_1_) and pyridoxal (vitamin B_6_) in *Plasmodium* and other organisms [11–13]. Up- and down-regulation of DXPS expression in *Arabidopsis thaliana* results in the commensurate changes in the level of the isoprenoid levels providing strong evidence that DXPS catalyzes the rate-determing step of NMVA in this organism [14] and, by extension, in others.

The focus of the present study is to define the active site pocket of DXPS from *Deinococcus radiodurans* by constructing a set of site-directed mutants and to elucidate the order of substrate binding using steady-state kinetic analysis, product inhibition with DXP, and a dead end inhibition with *β*-fluoropyruvate. This work fosters a better understanding of the DXPS-catalyzed reaction and will aid in the rational design of DXPS inhibitors for the treatment of malaria and other human diseases.

## Materials and Methods

### Materials

Thiamine pyrophosphate (TPP), pyruvate, D,L-glyceraldehyde-3-phosphate (GAP), 1-deoxy-D-xylulose-5-phosphate sodium salt, bovine serum albumin, and LB-broth were purchased from Sigma Aldrich. NADPH was purchased from Alexis Biochemical, Ni-NTA resin was purchased from Invitrogen, and β-mercaptoethanol (β-Me) was purchased from Fisher. *E. coli* XL-10 cells, deoxynucleotide mix PCR grade, *pfu*Ultra Hotstart DNA polymerase, QuikChange II site directed mutagenesis kit and acetonitrile (HPLC grade) were purchased from Agilent. The DNA vectors, *pET28a (+)* and *pET15b (+)*, and *E. coli* BL-21 B (DE3) cells were purchased from EMD Biosciences. DNA sequencing services and primers were purchased from MWG operon. All the other reagents were of the highest quality commercially available.

### Cloning of *D. radiodurans* DXPS and *E. coli* 1-Deoxy-D-xylulose 5-phosphate Reductoisomerase (DXR)

A synthetic, codon optimized *D. radiodurans dxps* gene with 5′-*NdeI* and 3′-*XhoI* restriction sites in a pMK vector was purchased from Geneart (Germany). The *dxps* gene was excised from the pMK vector and cloned into the *NdeI* and *XhoI* sites of a *pET28a (+)* vector (kanamycin resistance) with N-terminal His_6_-tag to yield the *pET28a (+)-pxps* plasmid. Successful cloning of the *D. radiodurans dxps* gene was confirmed by DNA sequencing at MWG Operon.

A synthetic, codon optimized *E. coli dxr* gene with 5′-*NdeI* and 3′-*BamHI* restriction sites in a pMK vector was purchased from Geneart (Germany). The *dxr* gene was excised from pMK vector and cloned into *NdeI* and *BamHI* restriction sites of *pET15b (+)* vector with a C-terminal His_6_ tag to yield the *pET15b (+)-dxr* plasmid. Gene insertion was confirmed by DNA sequencing.

### Production of the *D. radiodurans* DXPS mutants

Site-directed mutagenesis was carried out using the QuikChange II site-directed mutagenesis kit. Briefly, the mutagenesis mixture consists of 50–100 ng plasmid *pET28a (+)-dxps* as a template, 1X PCR reaction buffer, 0.4 mM each of the forward and reverse primer, 0.25 mM dNTP mixture, 5 μL Quik solution, and 2.5 units of *pfu* Ultra hot-start polymerase in a 50 μL reaction. The overlap extension method was used to produce the DXPS mutant that was difficult to create via site directed mutagenesis [15]. The sequence of the mutant DNA was confirmed by DNA sequencing.

### Over-expression and purification of Wildtype DXPS and the DXPS mutants

Plasmids containing the wild type *D. radiodurans dxps* gene or the mutant *dxps* gene were transformed into *E. coli* BL-21 B (DE3) cells and used for protein expression. An overnight culture of *E. coli* in LB broth containing 50 μg/mL *kanamycin* was diluted 100-fold, cultured at 37°C until the absorbance at 600 nm reached ~0.6, and then cooled to 20°C. Expression was induced by the addition of 0.5 mM isopropyl *β*-D-1-thiogalactopyranoside (IPTG). The cells were harvested by centrifugation (6,000 × *g* for 10 min) after being shaken for 6 hrs at 20°C and the resulting cell pellets stored at −80°C before purification. Cells were thawed and all the purification steps were performed at 4°C. Cells were resuspended in binding buffer (20 mM Tris, 500 mM NaCl, 5 mM imidazole, 10 mM *β-*mercaptoethanol (β-Me), pH=7.5) supplemented with 1 mM phenylmethanesulfonylfluoride (PMSF), 4 μg/mL leupeptin, and 2 μg/mL pepstatin, sonicated using a Heat systems W-380 ultrasonic processor, and centrifuged (16,000 × *g* for 20 min) to remove cell debris. The supernatant from the cell lysate was applied to a 1.5 cm × 5 cm column packed with Ni-NTA resin that had been equilibrated with binding buffer. Non-bound proteins eluted from the column by first washing with 5 column volumes of binding buffer followed by 20 column volumes of wash buffer (20 mM Tris pH 7.5, 500 mM NaCl, 60 mM imidazole, and 10 mM β-Me). The bound DXPS (wildtype or mutant) was eluted from the Ni-NTA resin using elution buffer (20 mM Tris pH 7.5, 500 mM NaCl, 250 mM imidazole, and 10 mM β-Me). A flow rate of 1.5 mL/min was maintained through the Ni-NTA column for all the loading and washing steps. DXPS-containing fractions containing were combined, exhaustively dialyzed at 4°C against 20 mM Tris pH 7.5, 100 mM NaCl, and 10 mM β-Me, and concentrated by ultrafiltration. The final yield of DXPS (wildtype or mutant) was 7–8 mg/L of *E. coli* culture medium. Enzyme was flash frozen in liquid nitrogen, stored at −80°C. The purity of the DXPS (wildtype or the mutant) was evaluated by SDS-PAGE.

### Determination of GAP and DXP concentration

D,L-GAP was obtained from Sigma Aldrich as a suspension in water. The concentration of D-GAP was measured spectrophotometrically using glyceraldehyde-3-phosphate dehydrogenase (GAPDH). A reaction mixture (0.5 mL) containing 30 mM sodium pyrophosphate buffer pH 8.6, 5 mM L-cysteine, 0.6 mM NAD^+^, 0.6 mM sodium arsenate, and D, L-GLP at concentration less than 0.2 mM were incubated at 30°C for 10 min, the reaction initiated with 2 μg of GAPDH (Novus Biotechnology), and the progress of the reaction monitored spectrophotometrically at 340 nm. The final concentration of D-GAP was calculated based on the production of NADPH (ε_340_=6,220 M^−1^ cm^−1^). Throughout this work, the abbreviation “GAP” represents the concentration of D-glyceraldehyde-3-phosphate in the racemic mix.

DXP was purchased from Sigma Aldrich as the sodium salt. The salt was resuspended in 10 mM HEPES buffer (pH=6.0), and the concentration was measured spectrophotometerically using DXR. A reaction mixture (0.5 mL) containing of 100 mM HEPES pH 8.0, 1 mg/ml BSA, 1.5 mM MnCl_2_, 0.3 mM NADPH, and DXP at concentration less than 0.1 mM were incubated at 37°C for 5 min, the reaction initiated with 1.25 μg of DXR, and the progress of the reaction monitored spectrophotometrically at 340 nm. Final concentration of DXP was calculated based on the oxidation of NADPH (ε_340_=6,220 M^−1^ cm^−1^).

### Assays for DXPS activity

One assay we employed to measure DXPS activity involved a DXPS-DXR coupled assay. In this way, the DXPS-dependent production of DXP is ultimately coupled to the oxidation of NADPH to NADP^+^. The solution for the DXPS-DXR coupled contained 100 mM HEPES pH 8.0, 100 mM NaCl, 1 mg/ml BSA, 1 mM TPP, 1.5 mM MnCl_2_, 2 mM β-Me, 0.15 mM NADPH, 0.2 mg/ml DXR, and varying concentrations of pyruvate or GAP [16]. Steady-state kinetic experiments were performed by varying pyruvate or GAP at a fixed saturating concentration of the co-substrate. A DXPS-DXR reaction solution was incubated at 37°C for 5 min, the reaction was initiated by addition of 358 nM DXPS, and the progress of the reaction monitored spectrophotometrically at 340 nm for the oxidation of NADPH.

Another assay we employed to measure DXPS activity was an HPLC determination of net [DXP] production or [GAP] consumption, modified from a published procedure [17]. This endpoint assay to measure DXPS activity was used in our product inhibition studies. Briefly, the reaction was initiated by addition of 573 nM DXPS. At the desired time, an aliquot (50 μL) was removed and added to an equal volume of acidic 2,4-dinitrophenol (DNP) (100 mM 2,4-DNP in 2N H_2_SO_4_) to terminate the enzymatic reaction. After a 5 minute incubation at room temperature, the pH was adjusted to 5–7 by addition of 36 μL 4.0 M Tris buffer pH 10.0 and centrifuged to remove excess 2,4-DNP. The DNP derivatives of pyruvate, GAP, and DXP were separated using a 5 μm Discovery^®^ C_18_ column (25 cm × 4.6 mm) developed using the following conditions: flow rate=1.5 mL/min; solvent A, 100 mM ammonium acetate pH 4.6, 0.05% TFA; solvent B, acetonitrile, 0.05% TFA; 20% – 35% solvent B over 12 min; and then 35–60% solvent B over 2 min. When varying [pyruvate] at one fixed [GAP], the concentrations used for the initial velocity kinetic experiments were as follows: 1.0 mM GAP and pyruvate at 0.26, 0.50, 0.75, or 4.0 mM; and DXP at 0, 0.2, 0.3, or 0.4 mM. The DXP hydrazone peak area was measured and the DXP concentration was determined against a standard curve generated using DXP hydrazone. When carrying the DXP inhibition studies, net [DXP] produced was determined by substrating background [DXP] at zero time (before the addition of enzyme) from the total [DXP] produced at the desired time interval. When varying [GAP] at one fixed [pyruvate], the concentrations used for the initial velocity kinetic experiments were as follows: 0.3 mM pyruvate and GAP at 0.05, 0.1, 0.25, and 0.50 mM; and DXP at 0, 0.2, 0.3, or 0.4 mM. The GAP hydrazone peak area was measured and the GAP concentration was determined compared against a standard curve generated using GAP hydrazone. Assays were performed in duplicate.

### Inhibition of DXPS by β-fluoropyruvate (F-Pyr)

Initial reaction velocities were determined using the DXPS-DXR coupled assay. When varying [pyruvate] at one fixed [GAP], the concentrations used for the initial velocity kinetic experiments were as follows: 0.5 mM GAP, pyruvate, 0.1 mM, 0.18 mM, 0.5 mM, or 2 mM; and F-Pyr at 0 μM, 25 μM, 50 μM, 75 μM or 100 μM. When varying [GAP] at one fixed [pyruvate], the concentrations used for the initial velocity kinetic experiments were as follows: 2.0 mM pyruvate and GAP at 0.025 mM, 0.05 mM, 0.125 mM, or 0.25 mM; and F-Pyr at 0, 25 mM, 50 mM, 75 mM or 100 μM. Assays were performed in triplicate using 115 nM of *D. radiodurans* DXPS and the data were fit using nonlinear least-squares regression analysis.

### Analysis of the initial velocity kinetic data

The steady state initial velocity for DXPS measured at various concentrations of pyruvate and GAP were fit to equation 1 using nonlinear regression analysis in Sigma-Plot 12.0 Data for inhibition of DXP and fluoropyruvate against pyruvate and GAP were fit to equation 2, 3 and 4 for competitive, noncompetitive, and uncompetitive, respectively, where [I] is the concentration of inhibitor and K_i_ is the inhibition constant with respect to *β*-fluoropyruvate.

(1) $$\nu =\frac{V\max[S]}{Km+[S]}$$

(2) $$\nu =\frac{V\max[S]}{Km(1+\frac{\left[I\right]}{Ki})+[S]}$$

(3) $$\nu =\frac{V\max[S]}{Km(1+\frac{\left[I\right]}{Ki})+[S](1+\frac{\left[I\right]}{Ki})}$$

(4) $$\nu =\frac{V\max[S]}{Km+[S](1+\frac{\left[I\right]}{Ki})}$$

## Results and Discussion

### Overexpression and purification of *D. radiodurans* DXPS

A synthetic, codon-optimized wildtype *D. radiodurans dxps* gene *(dxps-wt)* with a N-terminal His_6_ tag was cloned into the pET28a (+) vector using *NdeI* and *XhoI* restriction sites. A series of *D. radiodurans* DXPS mutant proteins were constructed near the active site pocket using the *dxps*-wt gene as template by the Quikchange site-directed mutagenesis method or the overlap extension method [15]. Wildtye enzyme (DXPS-WT) and the mutant enzymes (all possessing N-terminal His_6_ tag) were affinity purified using the Ni-NTA resin to >95% pure with respectable expression yields of 6–8 mg per liter of culture.

### Comparison of steady-state kinetic parameters between mutant DXPS proteins and wildtype enzyme

**Figure 2:**
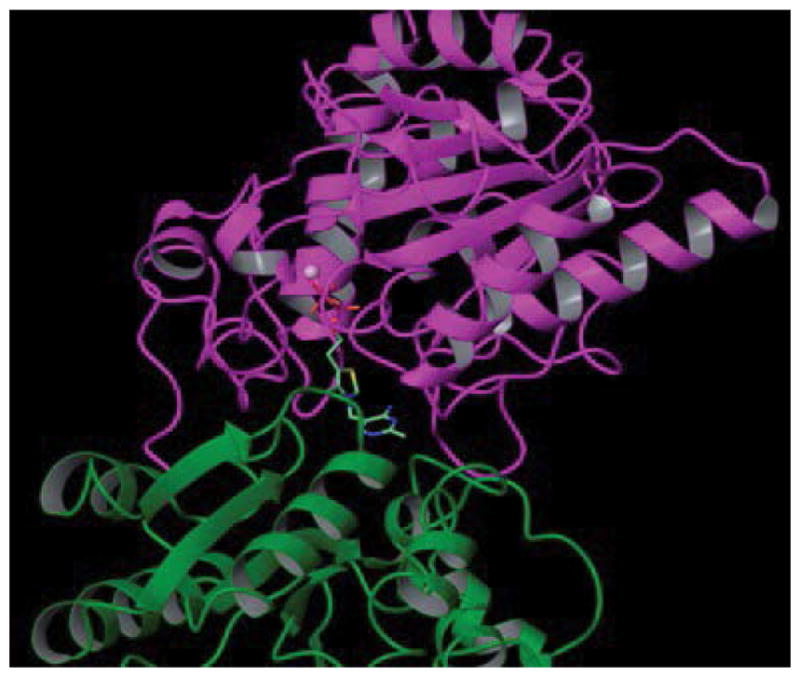
Schematic of *D. radiodurans* DXPS depicting the bound TPP. This figure was created using Maestro 9.3.023 from Schrödinger suite 2012 and PDB 2o1x. Domain I of *D. radiodurans* DXPS is shown as purple ribbon, domain II of *D. radiodurans* DXPS is shown as green ribbon as ribbon, TPP is shown as tube with aquamarine carbon atoms, and the Mg (II) ion is shown as a pink sphere.

**Figure 3:**
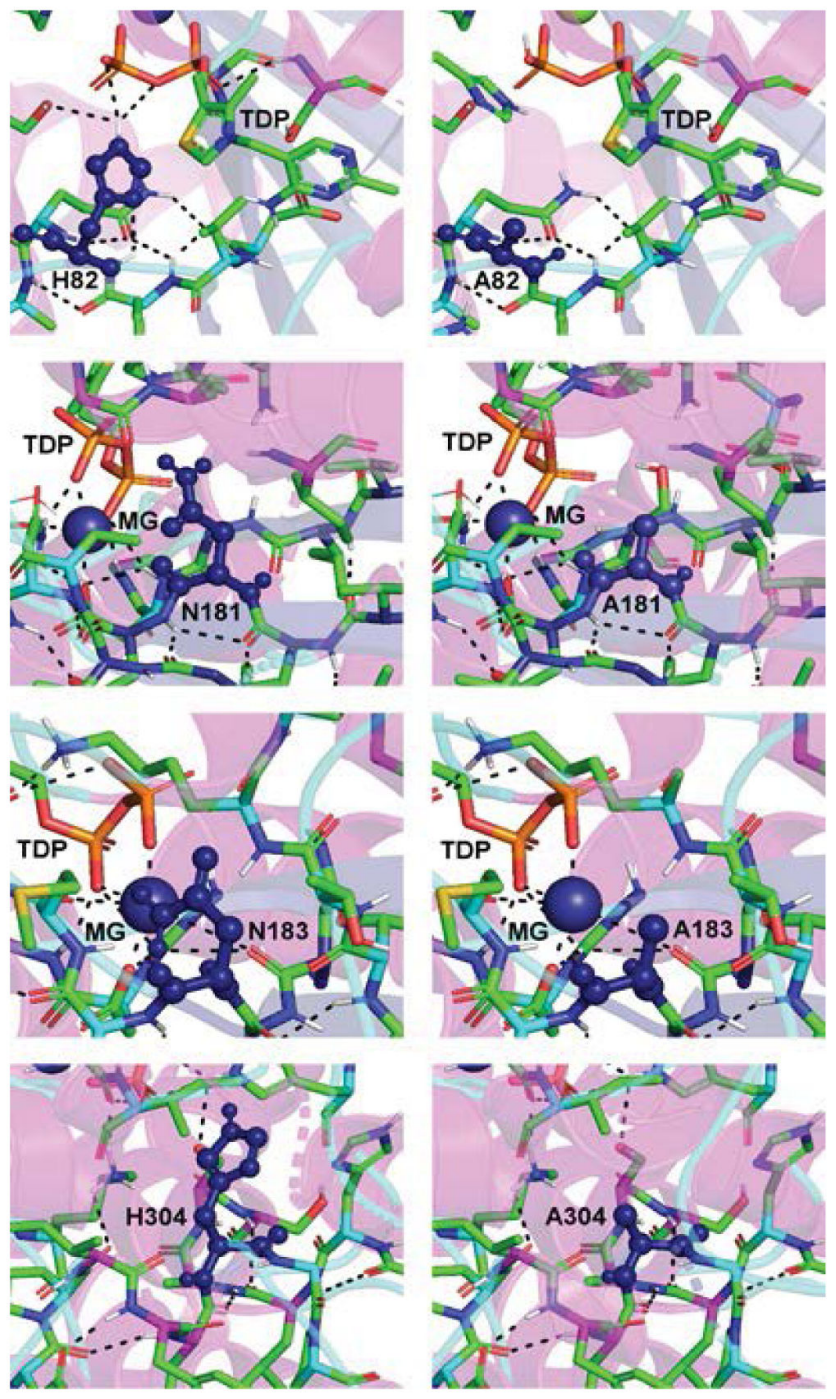
Domain I of *D. radiodurans* DXPS including the bound TPP molecule. Domain I mutant enzymes (H82A, N181A, N183, and H304A) with the dashed lines representing hydrogen bonds and key residue highlighted in deep blue. The models in the column on the left show the wild-type enzyme while the mutant enzymes are shown in the models in the column on the right. These models do not represent all possible differences in hydrogen bonding due to the lack of substrates in the active but, no crystal structures of DXS currently exist containing the bound. The models were prepared utilizing PyMOL.

**Figure 4:**
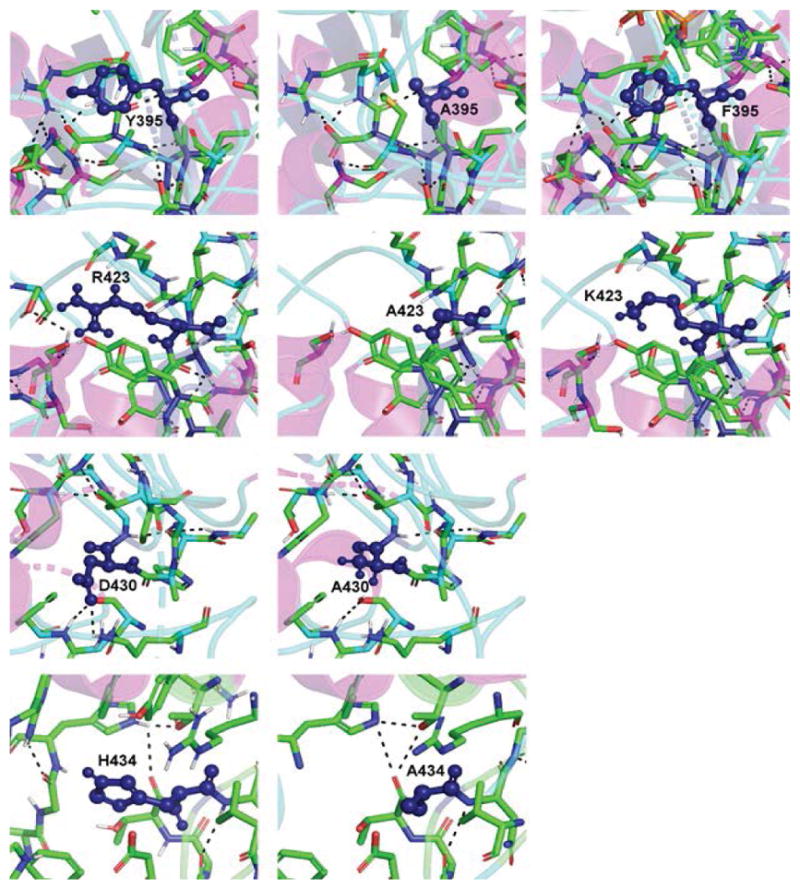
Domain II of *D. radiodurans* DXPS including the bound TPP molecule. Domain II mutant enzymes (Y395A, Y395F, R423A, R423K, D430A, and the H434A) with the dashed lines representing hydrogen bonds and key residue highlighted in deep blue. The left column on the far left is a model for the wild-type enzyme while the models in the right column images illustrate the mutant enzymes. The top 2 rows are the three models that correlate with the Y395 and R423 mutants, respectively. The models were prepared utilizing PyMOL.

We have constructed and evaluated a set of mutant *D. radiodurans* DXPS proteins. DXPS is a TPP- and Mg (II)-dependent enzyme that belongs to the transketolase (TK) family of TPP-dependent enzymes [18,19]. The sequences of *E. coli* and *D. radiodurans* DXPS are 45% identical and their respective structures are remarkably similar. The rms distance of equivalent C_α_ atoms between the two structures is only 0.7Å [19]. Despite low sequence identity to other TK family members, *E. coli* and *D. radiodurans* structures are similar to that of yeast transketolase (TK), the E1 subunit of *E. coli* pyruvate dehydrogenase (PDH), and *P. putida* 2-oxoisovalerate dehydrogenase [19,20]. DXPS is a dimer, with each monomer possessing three domains: domains I, II, and II (Figure 2). Domain I is composed of residues 1–319, domain II is composed of residues 320–495, and domain III is composed of residues 496–629. The DXPS active site is found at interface of domains I and II within the same monomer (Figures 3 and 4) [19,20]. In constrast to DXPS, the active site of other TPP-dependent enzymes is located at the interface of domain I of one monomer and domain II of the second monomer, examples include the E1 component of *E. coli* pyruvate dehydrogenase [21], yeast transketolase [22], yeast acetohydroxyacid synthase [23], *Bifidobacterium longum* phosphoketolase [24]. TPP is bound deeply within a cleft formed from segments of domain I and II and adopts a V-conformation, like that found in other TPP-dependent enzymes [19]. The V-conformation of TPP positions the 4′-imino group to deprotonate the C2 atom, thus, forming the catalytically important C2 carbanion [25,26]. Sequence alignments of DXPS proteins from a number of different organisms show extensive sequence identity at the binding sites for TPP and GAP [19,20].

### Domain I mutants

A structural feature common too many TPP-dependent enzymes is the presence of three discrete domains, as was found for DXPS. The Pyr domain interacts with the pyrimidine ring of the TPP cofactor and PP domain interacts with the pyrophophosphate tail of TPP. The function of the third domain is unclear, but may serve a regulatory function as this domain is structurally related to nucleotide binding domains in other proteins [27]. As reviewed by Duggleby [27], the relative arrangement of the three domains is different within the various families of the TPP-dependent enzymes. Domain I of *D. radiodurans* DXPS is the PP domain and functions primarily in the binding of the pyrophosphate moiety of TPP. The DXPS-bound Mg (II) is coordinated to the TPP pyrophosphate, meaning the amino acids necessary for Mg (II) binding are found within domain I. GAP is bound within a pocket that is composed of amino acids from both domains I and II; thus, domain I also has a role in GAP binding.

The results for the domain I mutants constructed herein are shown in Tables 1 and 2. His-82 and His-304 are two amino acid residues involved in TPP binding: the N3 imidazole His-82 is 5.6Å from C2 of the thiazolium ring and the N1 of the imidazole of His-304 is 5.2Å from the C2 of the thiazolium ring. Also, the N1 of the imidazole is 4.1Å from N1 of the imidazole of His-304 in *D. radiodurans* DXPS. The mutation of both to Ala, generating separate H82A and H304A mutant proteins, yielded catalytically defective enzymes with V_max_ and (V/K)_app_ values of 2–12% of wild-type. Given the proximity of His-82 and His-304 in the *D. radiodurans* DXPS structure, it seems likely that these amino acids could function “in reserve” of each other accounting for the ~10% remaining in both H82A and H304A mutants. In other words, His-304 can partially fulfill the role played by His-82 in catalysis after its mutation to Ala.

The K_M,app_ values for the H82A mutant for both GAP and pyruvate are similar to wildtype indicating that His-82 contributes little to the binding of either substrate. Similar to our results for the H82A mutant, mutation of the equivalent amino acid in yeast transketolase, His-69, to Ala yielded an enzyme with a V_MAX_ that is 1.5% of wildtype and virtually the same K_M_ for the acceptor substrate (ribose 5-phosphate for transketolase and GAP for DXPS). In contrast to our results for H82A, the donor substrate K_M_ in yeast transketolase does increase 6-fold [28]. These data indicate that His-82 and His-69 serve to stabilize the α-carbanion/enamine-TPP intermediate produced from the donor substrate in DXPS and transketolase, which was observed later in the structure of the yeast transketolase-*α*, *β*-dihydroxyethyl-TPP complex [29]. One difference between the two enzymes is the role served by His-82 or His-69 in donor substrate binding. In *D. radiodurans* DXPS, His-82 seems to have no role in donor substrate binding while in yeast transketolase, His-69 does contribute to donor substrate binding.

The results for the H304A mutant differed from that of H82A as the K_M,app_ for GAP was similar to wildtype, but the K_M,app_ value for pyruvate was 6-fold higher than wildtype. The structure of *D. radiodurans* DXPS shows that His-304 is located within an open cavity near the thiazolium C2 atom and, thus, likely contributes to both pyruvate binding and stabilization of the α-carbanion/enamine-TPP intermediate.

Other mutations that were constructed within domain I were N181A and N183A. Asn-183 is coordinated to the bound Mg (II) ion and mutation of this residue to Ala results in a completely inactive protein. In contrast to N183A, the N181A mutant retains activity exhibiting 10% of (V/K)_app,GAP_ and 70% of (V/K)_app,pyruvate_. The K_M,app_ values for both substrates are approximately the same as wildtype for the N181A mutant; thus, the decrease in (V/K)_app_ reflects a decrease in catalytic efficiency upon mutant of Asn181 to Ala. The amino acid equivalent to Asn-181 in yeast transketolase is Asp-185. The structure of yeast transketolase showns that Asp-185 is involved in a network of bound water molecules important in Ca (II) chelation and pyrophosphate binding in this enzyme. Asn-181 may serve a similar role in *D. radiodurans* DXPS. A mutation in this residue could subtly alter the active site architecture such that the TPP is no longer optimally positioned for catalysis. Models of the domain I mutants illustrating the critical active site amino acids and hydrogen bonds are shown in Figure 3.

### Domain II mutants

Amino acids 320–495 of *D. radiodurans* DXPS define domain II, which primarily interacts with the thiazolium and aminopyrmidine rings of TPP. Thus, domain II in DXPS is the Pyr domain [27]. Domain II also serves an important role in the binding of both substrates, GAP and pyruvate [19,20]. Of the amino acids in domain II that we altered via mutagenesis, the largest effects on catalysis were observed in changing Arg-423 (Tables 3 and 4). Arg-423 is conserved in other DXPS enzymes and has a role in GAP binding resulting from the formation of hydrogen bond between its guanidino group and the phosphate of GAP. Mutation of Arg-423 to a lysine yields an enzyme with considerable residual activity (50–80%), but with altered K_M_ values; the K_M,GAP_ increasing 12-fold and the K_M,pyruvate_ decreasing 5-fold. Mutation of Arg-423 to an alanine yields a mutant enzyme with little activity, a (V/K) _app_ that is 0.1% of wildtype. The K_M,GAP_ for the R423A mutant is approximately equivalent to the K_M_ for glyceraldehyde in DXPS WT (14 mM), providing additional evidence that the guanidino moiety of Arg-423 interacts with the phosphate of GAP. The amino acid equivalent to Arg-423 in *D. radiodurans* DXPS in yeast transketolase is His-469. The structure of yeast transketolase shows that imidazole of His-469 forms a hydrogen bond with the phosphate of the acceptor substrate (erythrose 4-phosphate) [30] and, in addition, may form a similar hydrogen bond to the phosphate in the substrate-TPP adduct [31]. In contrast to the R423 mutant described herein, the yeast transketolase H469A mutant retains considerable activity, but exhibits ~10-fold higher K_M_ values for both substrates. While Arg-423 and His-469 have key roles in the binding of the phosphorylated substrates in both DXPS and transketolase, the positive charge of Arg-423 is more important to catalysis in DXPS than that of His-469 in transketolase.

Modeling studies suggest that the hydroxyl group of Tyr-395 interacts directly with the phosphate of GAP, yet it has been reported that the mutation of the equivalent tyrosine residue in *E. coli* DXPS, Tyr-392, has little effect on catalysis [19]. Both the Y392A and Y392F mutants of *E. coli* DXPS are more active than wildtype enzyme. We find that mutation of Tyr-395 in *D. radiodurans* DXPS does compromise catalytic efficiency as both the Y395A and Y395F mutant exhibit k_cat_/K_M_ values that are only >10% of that of wildtype (Table 1) ;or GAP. The decrease in catalytic efficiency in both the Y395A and Y395F mutants is largely reflected in a >10-fold increase K_M_ for both proteins. The Y395F mutant is a better catalyst than the Y395A mutant: (k_cat_/ K_M_)_Y395F_/(k_cat_/K_M_)_Y395A_=1.7, indicating that the aromatic ring and hydroxyl group of Y395 contribute positively to catalysis. This is consistent with studies of the amino acid equivalent of Tyr-395 in yeast transketolase, Phe-442, showing that aromatic side chain of Phe-442 contributes to a hydrophobic binding pocket for the pyridine ring of the TPP cofactor [31].

Like Tyr-395, Asp-430 is thought to interact with GAP, in this case, forming a hydrogen bond to the hydroxyl group at carbon-2. The equivalent amino acid in yeast transketolase, Asp-477, similarly forms a hydrogen bond to the 2-hydroxyl group of erythrose 4-phosphate [30]. Mutagenesis of Asp-430 to alanine yields a mutant enzyme that retains considerable activity, with a V/K that is ~50% of wildtype, resulting from a ~2-fold increase in the K_M_ for both substrates (Table 1). Construction of the similar mutant in yeast transketolase, D477A, yielded a protein of much lower activity, 1–2% of wildtype, with relatively greater values for the substrates. The K_M_ for the donor substrate in the D477A mutant, xylulose 5-phosphate, increased 9-fold while that for the acceptor substrate, ribose 5-phosphate, increased 12-fold [30]. In the reaction catalyzed by DXPS, pyruvate is the donor substrate and GAP is the acceptor substrate. These data suggest that Asp-477 in yeast transketolase has a more important role in acceptor substrate binding and catalysis that Asp-430 in *D. radiodurans* DXPS.

The amino acid equivalent of His-434 in *D. radiodurans* DXPS is His-481 in yeast transketolase and Gln-428 in human transketolase [32]. Mutation of Gln-428 to Ala, His, Asn, or Glu in human transketolase yields an enzyme that retains activity with the V_max_ values ranging from 5% of wildtype for Q428E to 18% of wildtype for Q428N. More detailed analysis Q428 shows no difference in the K_M_ values relative to wildtype for either the donor or acceptor substrates, but with a decrease in V_MAX_ to 15% of wildtype. These results suggested that Gln-428 was not important to substrate binding in human transketolase, but was important to catalysis. Singleton et al. suggested that Gln- 428 aids in orienting an active site water molecule critical to the optimum positioning of TPP [32]. Mutagenesis studies of His-481 in yeast transketolase differed from what was found for Gln-428 in the human enzyme. Conversion of His-481 to Ala, Ser, or Gln produced of lower activity, with the V_max_ values all ≤ 5% of wildtype. Steady-state kinetic analysis of the yeast transketolase mutants showed no effect on the K_M_ for the acceptor substrate, but a large increases in the K_M_ value for the donor substrate, ranging from an 18-fold increase for H481A to 58-fold for the H481Q [28]. Crystallographic studies of yeast transketolase in the presence of β-hydroxypyruvate only, which will form the enzyme bound α,β-dihydroxyethyl-thiamin intermediate, shows that His- 481 interacts with the donor substrate and forms a stabilizing hydrogen bond from the N1 of the imidazole ring to the C2 hydroxyl of this intermediate [29].

Analysis of the H434A mutant of *D. radiodurans* DXPS yielded results that differed from what has been reported for human and yeast transketolases. The V_max_ of the H434 mutant is higher than that of wildtype, V_max_ (H434)/V_max_ (wildtype)=1.2 – 1.3, and the K_M_ values for both the donor and substrates in the H434 mutant are also increased approximately the same relative to wildtype. Mutagenesis of the equivalent amino acid in *E. coli* DXPS, His-431, to alanine produced an enzyme with the same activity as wildtype [19], consistent with our data on the *D. radiodurans* enzyme. The K_M_ for GAP increased 4-fold while the K_M_ for pyruvate increased 6-fold (Table 3). Modeling studies of *D. radiodurans* DXPS suggest that His-434 is located with the GAP binding pocket without an obvious direct interaction with the substrate. The negatively charged phosphate of GAP seems to dock into a positively charged well in the active site and His-434 seems to contribute to this positively charged active site well. Based on the model of pyruvate-TPP adduct in *D. radiodurans* DXPS, it is conceivable that pyruvate may also interact with this postively charged well in the active site. Replacement of His-434 with alanine will decrease the net positive charge of the well and increasing the K_M_ for both substrates with little effect on the rate of catalysis. Models of the domain II mutants illustrating the critical active site amino acids and hydrogen bonds are shown in Figure 4.

### Inhibition and kinetic studies

The kinetic mechanism has been investigated for many TPP-dependent enzymes; a classic ping-pong scheme is common within this family of enzymes [33–35]. A ping-pong kinetic mechanism means that first substrate binds followed by the release of the first product before the second substrate binds. DXPS is an unusual TPP-dependent enzyme as substrates, pyruvate and GAP, bind before pyruvate is decarboxylated; thus, the kinetic mechanism for DXPS is not ping-pong [36–39]. Consistent with this unusual aspect of DXPS-mediated catalysis is the incredible stability of the C2-α-lactyl-TPP intermediate within the active site [38]. The binding of GAP to form the E•C2-α-lactyl- TPP•GAP ternary complex triggers CO_2_ release, yielding the C2- α-carbanion/enamine that will react with GAP to produce DXP [38,39]

Dead-end inhibition studies and ^14^CO_2_ trapping experiments demonstrated that the *Rhodobacter encapsulates* DXPS has an ordered sequential mechanism with pyruvate binding first [36]. Eubanks and Pouter [36] found that the conversion of pyruvate to ^14^CO_2_ is inefficient in absence of GAP, consistent with stability of the DXPS-bound C2-α-lactyl-TPP intermediate. Studies of *E. coli* DXPS showed that the kinetic mechanism for this enzyme was rapid equilibrium random; pyruvate and GAP binding to *E. coli* DXPS is reversible and completely independent of each other [37–39].

**Figure 5:**
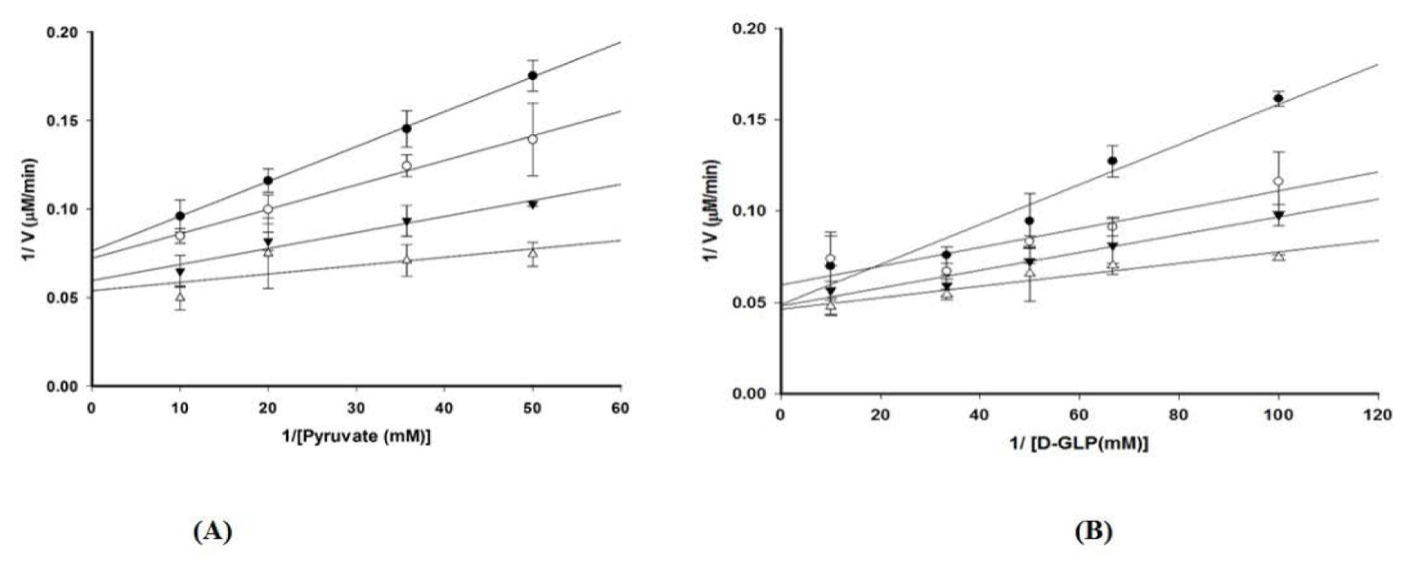
Double reciprocal analysis of initial velocities of pyruvate and GAP at different fixed concentrations of the other substrate. Right panel: kinetic analysis was performed using sub-saturating concentrations of GAP: 0.01 mM (●), 0.014 mM (○), 0.02 mM (▼), and 0.03 mM (△). Left panel: kinetic analysis was performed using subsaturating concentration of pyruvate: 0.02 mM (●), 0.03 mM (○), 0.04 mM (▼), and 0.06 mM (△).

In our study of *D. radiodurans* DXPS, we employed a set of steady-state kinetic experiments by varying the concentration of one substrate and at constant, but different concentrations of the other substrate. Results from these experiments were coupled with product (DXP) inhibition data and dead-end inhibition data (*β*-fluoropyruvate) to provide information regarding the kinetic mechanism. Double reciprocal analysis at lower concentrations of pyruvate (0.02 mM - 0.06 mM) (Figure 5A) and GAP (0.01 – 0.03 mM) (Figure 5B) at different, but fixed concentrations of the other substrate revealed a non-competitive pattern with change in both slope and intercept, eliminating a ping-pong and rapid equilibrium ordered kinetic mechanism [40]. The lack of curvature in the plots at high substrate concentration further suggests that the kinetic mechanism for *D. radiodurans* DXPS is not steady-state random [40,41].

**Figure 6:**
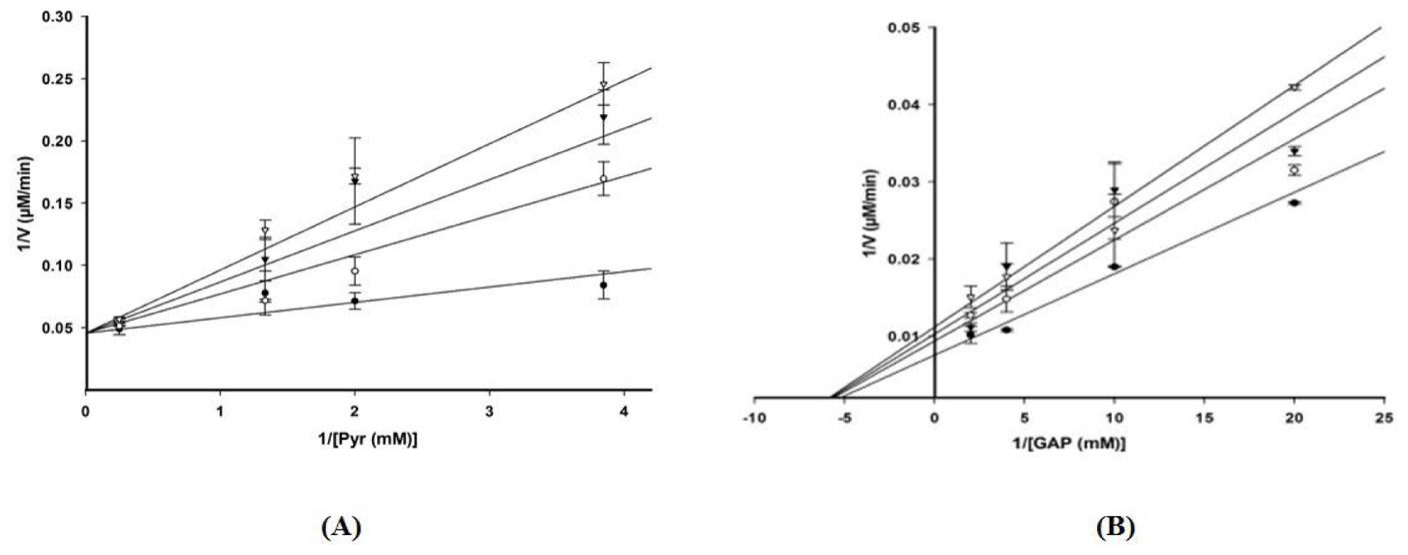
Double reciprocal analyses of initial velocities of DXPS inhibition by DXP at different initial concentrations of pyruvate (right panel) and GAP (left panel). Kinetic analyses were performed using the following concentrations of DXP: 0 mM (●), 0.2 mM (○), 0.3 mM (▼) and 0.4 mM (▽).

**Figure 7:**
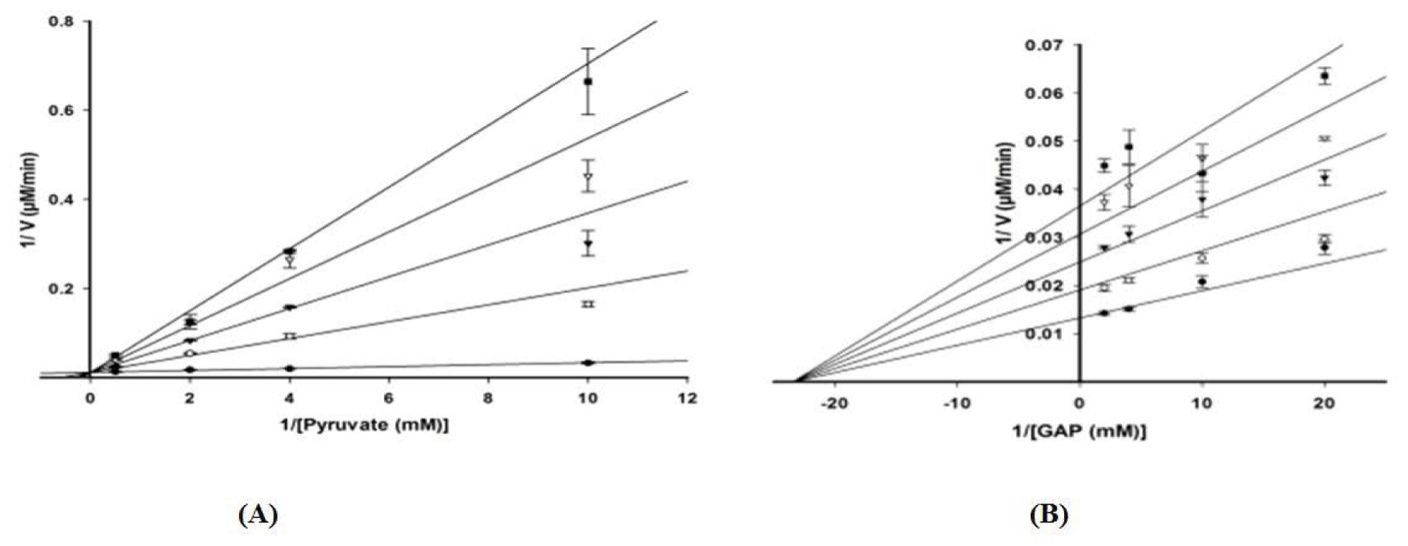
Double reciprocal analyses of initial velocities of DXPS inhibition by F-Pyr at different fixed concentration of pyruvate (right panel) and GAP (left panel). Kinetic analysis was performed using the following concentrations of F-Pyr: 0 μM (●), 25 μM (○), 50 μM (▼), 75 μM (▽), and 100 μM (■).

**Figure 8:**
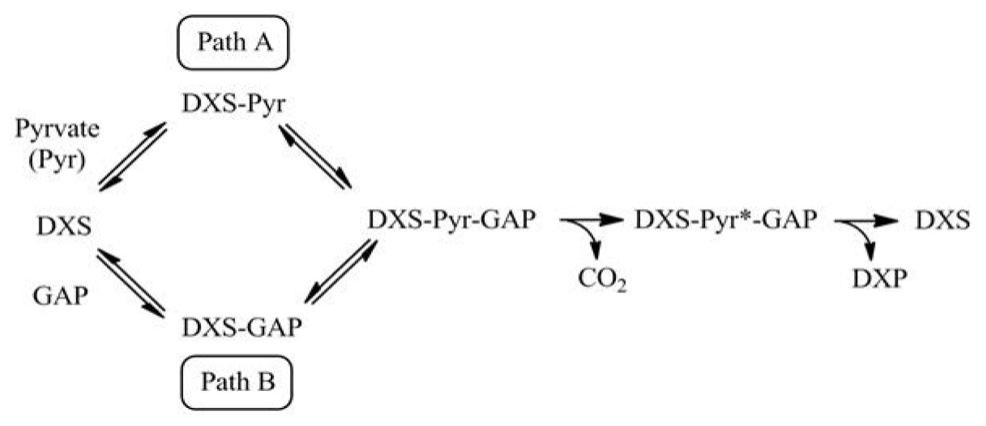
The random sequential pathway proposed for the reaction catalyzed by *D. radiodurans* DXPS. Pyr* is the C2-α-carbanion/enamine intermediate as shown in Figure 1.

Product inhibition studies were next used to determine the mechanism of substrate binding and product release in the *D. radiodurans* DXPS catalyzed reaction. DXP was used as product inhibitor against pyruvate and GAP. We found that DXP is a weak inhibitor with ~60% inhibition at concentration of 0.4 mM, yielding a K_i_ in low millimolar range (Table 5). DXP showed a competitive pattern vs. pyruvate indicating that DXP competes for the same enzyme form as pyruvate (Figure 6A), despite the chemical resemblance between DXP and GAP. When GAP was the varied substrate at fixed sub-saturating concentration of pyruvate, DXP yielded a non-competitive inhibition pattern (Figure 6B). This shows that DXP and GAP do not bind to the same enzyme form. At a saturating concentration of pyruvate, no inhibition was detected by DXP when GAP was varied substrate. On the other hand, DXP inhibition was still observed at saturating concentration of GAP when pyruvate was the variable substrate. F-Pyr was used as a dead-end inhibitor to further study the order of substrate binding in *D. radiodurans* DXPS. F-Pyr is competitive vs. pyruvate (Figure 7A) and non-competitive vs. GAP (Figure 7B) and exhibits a K_i_=3.3 μM when pyruvate was the variable substrate. In sum, all the data presented herein, (a) the DXP inhibition patterns, (b) the double reciprocal plots obtained in varying both substrates, and (c) the F-Pyr inhibition point to either a Theorell-Chance ordered, steady-state ordered, or rapid equilibrium random kinetic mechanism. A Theorell- Chance kinetic mechanism, a scheme that requires the lack of a kinetically significant E•C2-α-lactyl-TPP•GAP ternary complex, seems unlikely given the results of Brammer-Basta et al. [39] for *E. coli* DXPS and is not commonly found as a kinetic mechanism for TPP-dependent enzymes. Mostly likely kinetic mechanism for *D. radiodurans* DXPS is rapid equilibrium random. Note that a random mechanism could evolve to an ordered mechanism at high [pyruvate], similar to that reported for *R. capsulatus* DXPS [36]. At high [pyruvate], the *D. radiodurans* DXPS-catalyzed conversion of pyruvate and GAP to DXP and CO_2_ would proceed preferentially through path A relative to path B (Figure 8).

## Conclusion

We have produced and characterized a series of *D. radiodurans* DXPS mutants, the selection based upon the sequence alignment of a number of DXPS enzymes and relationships to key amino acids in other TPP-dependent enzymes. Mutant proteins were constructed of these domain I amino acids, His-82, Asn-181, Asn-183, and His-304, and these of domain II, Tyr-395, Arg-423, Asp-430, and His-434. The results of our mutation studies point to differences in the roles for Arg-423, Asp-430, and His-434 in substrate binding and catalysis for *D. radiodurans* DXPS relative to the equivalent amino acids in transketolase, another TPP-dependent enzyme. Since Arg-423, Asp-430, and His-434 are all found within domain II in *D. radiodurans* DXPS our data point towards broad differences in the role of domain II in substrate binding and catalysis in DXPS relative to transketolase. Another outcome from our mutagenesis work on *D. radiodurans* DXPS is identification of a potential “back-up” role between His-82 and His-304 in catalysis meaning that one of these His residues can fulfill the catalytic role of the other when the other His is converted to Ala by mutagenesis. We complemented our mutagenesis studies with experiements demonstrating that the kinetic mechanism of *D. radiodurans* DXPS is, most likely, rapid equilibrium random similar to that reported for *E. coli* DXPS. DXPS catalyzes the first step in the non-mevalonate pathway of isoprenoid biosynthesis in many pathogens. DXPS and the other enzymes of this pathway represent drug targets as these enzymes have no homology the enzymes of the mevalonate-dependent pathway of isoprenoid biosynthesis in man and other mammals. The work presented herein contributes to our understanding of TPP-dependent catalysis and provides information about the active site architechure and order of substrate binding for *D. radiodurans* DXPS. These data should aid in the rational design of DXPS inhibitors that to attack pathogens reliant on non-mevalonate pathway for their isoprenoids.

## Figures and Tables

**Table 1:** Domain I mutants: Measurement of steady-state kinetic parameters with GAP is the variable substrate^a^.

Protein	K_M,app_ (mM)	*k*_cat,app_ (s^−1^)	(*k*_cat_/K_M_)_app_ (s^−1^ M^−1^)	$\frac{{\left(k_{\text{cat}}/K_{M}\right)}_{\text{app}}}{{\left[{\left(k_{\text{cat}}/K_{M}\right)}_{\text{app}}\right]}_{\text{WT}}}$
Wildtype	0.05 ± 0.01	7.9 ± 0.4	1.5 × 10^5^	1.0
H82A	0.03 ± 0.01	0.37 ± 0.02	1.3 × 10^4^	0.09
N181A	0.03 ± 0.01	0.43 ± 0.02	1.4 × 10^4^	0.09
N183A	0	0	0	0
H304A	0.08 ± 0.02	0.90 ± 0.1	1.1 × 10^4^	0.07

a Apparent kinetic constants were measured by varying the initial concentration of GAP at an initial fixed concentration of 2.0 mM pyruvate.

**Table 2:** Domain I mutants: Measurement of steady-state kinetic parameters with pyruvate is the variable substrate^a^.

Protein	K_M,app_ (mM)	*k*_cat,app_ (s^−1^)	(*k*_cat_/K_M_)_app_ (s^−1^ M^−1^)	$\frac{{\left(k_{\text{cat}}/K_{M}\right)}_{\text{app}}}{{\left[{\left(k_{\text{cat}}/K_{M}\right)}_{\text{app}}\right]}_{\text{WT}}}$
Wildtype	0.28 ± 0.03	7.4 ± 0.3	2.6 × 10^4^	1.0
H82A	0.23 ± 0.02	0.38 ± 0.01	1.7 × 10^3^	0.07
N181A	0.17 ± 0.02	3.3 ± 0.1	1.9 × 10^4^	0.7
N183A	0	0	0	0
H304A	1.7 ± 0.5	0.90 ± 0.01	5.8 × 10^2^	0.02

a Apparent kinetic constants were measured by varying the initial concentration of pyruvate at an initial fixed concentration of 0.5 mM GAP.

**Table 3:** Domain II mutants: Measurement of steady-state kinetic parameters with GAP is the variable substrate^a^.

Protein	K_M,app_ (mM)	*k*_cat,app_ (s^−1^)	(*k*_cat_/K_M_)_app_ (s^−1^ M^−1^)	$\frac{{\left(k_{\text{cat}}/K_{M}\right)}_{\text{app}}}{{\left[{\left(k_{\text{cat}}/K_{M}\right)}_{\text{app}}\right]}_{\text{WT}}}$
Wildtype	0.05 ± 0.01	7.9 ± 0.4	1.5 × 10^5^	1.0
Y395A	0.67 ± 0.05	3.9 ± 0.1	5.8 × 10^3^	0.04
Y395F	0.59 ± 0.04	5.9 ± 0.2	1.0 × 10^4^	0.07
D430A	0.12 ± 0.01	7.7 ± 0.2	6.6 × 10^4^	0.4
R423A	12 ± 3.2	1.7 ± 0.3	1.4 × 10^2^	9 × 10^−4^
R423K	0.60 ± 0.1	6.4 ± 0.2	1.1 × 10^4^	0.07
H434A	0.23 ± 0.01	9.6 ± 0.3	4.2 × 10^4^	0.3

a Apparent kinetic constants were measured by varying the initial concentration of GAP at an initial fixed concentration of 2.0 mM pyruvate.

**Table 4:** Domain II mutants: Measurement of steady-state kinetic parameters with pyruvate is the variable substrate^a^.

Protein	K_M,app_ (mM)	*k*_cat,app_ (s^−1^)	(*k*_cat_/K_M_)_app_ (s^−1^ M^−1^)	$\frac{{\left(k_{\text{cat}}/K_{M}\right)}_{\text{app}}}{{\left[{\left(k_{\text{cat}}/K_{M}\right)}_{\text{app}}\right]}_{\text{WT}}}$
Wildtype	0.28 ± 0.03	7.4 ± 0.3	2.6 × 10^4^	1.0
Y395A	0.16 ± 0.01	3.6 ± 0.1	2.2 × 10^4^	0.8
Y395F	0.19 ± 0.01	2.9 ± 0.02	1.6 × 10^4^	0.6
D430A	0.52 ± 0.03	7.2 ± 0.2	1.4 × 10^4^	0.5
R423A	ND^b^	ND^b^	ND^b^	ND^b^
R423K	0.06 ± 0.01	3.9 ± 0.1	6.5 × 10^4^	2.5
H434K	1.7 ± 0.1	9.9 ± 0.2	5.9 × 10^3^	0.2+

a Apparent kinetic constants were measured by varying the initial concentration of GAP at an initial fixed concentration of 0.5 mM GAP.

b ND = Not Determined

**Table 5:** Inhibition patterns and inhibition constants for the inhibition of *D. radiodurans* DXPS by DXP or β-fluoropyruvate.

Inhibitor	Substrate	Pattern	K_i_ (μM)
DXP	Pyruvate	Competitive	130 ± 32
DXP	GAP	Non-competitive	830 ± 160
F-Pyr	Pyruvate	Competitive	3.3 ± 0.4
F-Pyr	GAP	Non-competitive	57 ± 3.2
